# A High-Performance, Low-Cost, and Integrated Hairpin Topology RF Switched Filter Bank for Radar Applications

**DOI:** 10.3390/s24020434

**Published:** 2024-01-10

**Authors:** Talha Shahid Alvi, Muhammad Haris Ahsan, Muhammad Ali, Faizan Ramzan, Khaled A. Aljaloud, Ali H. Alqahtani, Rifaqat Hussain, Akram Alomainy, Muhammad Qasim Mehmood

**Affiliations:** 1 MicroNano Lab, Department of Electrical Engineering, Information Technology University of the Punjab, Lahore 56000, Pakistan; bsee18018@itu.edu.pk (T.S.A.); bsee18017@itu.edu.pk (M.H.A.); msee20016@itu.edu.pk (M.A.); msee20021@itu.edu.pk (F.R.); 2 College of Engineering, Muzahimiyah Branch, King Saud University, P.O. Box 2454, Riyadh 11451, Saudi Arabia; kaljaloud@ksu.edu.sa (K.A.A.); ahqahtani@ksu.edu.sa (A.H.A.); 3 Antenna and Electromagnetics Research Group, School of Electronic Engineering and Computer Science, Queen Mary University of London, London E1 4NS, UK; rifaqat.hussain@qmul.ac.uk (R.H.); a.alomainy@qmul.ac.uk (A.A.)

**Keywords:** bandpass filter, insertion loss, return loss, switched filter bank

## Abstract

Switched filter banks find widespread application in frequency-hopping radar systems and communication networks with multiple operating frequencies, especially in situations demanding elevated filter element isolation. In this paper, the design and implementation of a highly isolated switchable narrow-bandpass filter bank architecture using hairpin microstrip topology is presented. The filter bank has four discrete bandpass filters with passbands of 2.0–2.2 GHz, 2.3–2.5 GHz, 3.1–3.3 GHz, and 3.9–4.1 GHz. These filters span the radar S-frequency band (2.0–4.0 GHz). In order to switch between channels with a switching speed of nanoseconds, low-loss and highly isolated SP4T switches are implemented. Advanced design system (ADS) software is used to design the various filter functionalities, and the entire system is tested on a vector network analyzer (VNA). The proposed architecture makes it much easier to put the filter bank into practice and switch it to the desired frequency, which is useful for radar receiver applications.

## 1. Introduction

Radar technology holds significance, as it enables the detection and monitoring of objects and substances from a distance, even in challenging visibility conditions. Its applications span various fields, such as aviation, military operations, meteorology, and law enforcement. For instance, in aviation, radar plays a crucial role in aircraft detection, tracking, and air traffic control. Additionally, in meteorology, radar is employed to identify and monitor precipitation, aiding meteorologists in predicting storms and issuing severe-weather warnings [1,2,3]. Its operation involves the exclusion of unwanted frequencies and the execution of a post-processing sequence on the corresponding signal. Filters play a vital role in carrying out the essential task of eliminating unnecessary harmonics [4,5,6]. The acquired wideband signals contain an inflated signal-to-noise ratio (SNR), which in return impacts the harmonics and data rate for channel capacity, diminishing the signal’s effectiveness. Devices that can extract narrow-band signals and improve the SNR and information data rate in addition to a white Gaussian noise channel are becoming a necessity for the system. In recent RF and microwave communications systems, narrow-band technology is widely used as the consequence of narrow-band potential [7]. Narrow-band systems have a very wide range of applications in sub-6 GHz space and in numerous devices, including filters, antennas, and other RF circuits. At microwave frequencies, microstrip technology is optimal for designing filters and antennas [8,9,10]. Designing a narrow-band filter for microwave frequencies is challenging, as it must filter out signals with low bandwidth and attain a sharp attenuation rate at the frequency band edges. In a narrow-band frequency signal, harmonic reduction is also challenging. Through the use of a microstrip-based narrow-band filter, these counterfeit effects can be diminished [11,12,13]. A low insertion loss, high return loss, and sharp roll-off rate are required from these microstrip-based filters. Various techniques are used to implement microstrip filters.

A vast body of literature explains the process of reducing harmonics in narrow-band signals using devices like filters. In the realm of microwave frequencies, microstrip filters prove to be optimal, offering maximum efficiency and high performance while maintaining low cost and compact size. Effective mechanisms within narrow-band filters are essential for blocking any interference in the signal. These filters serve as fundamental components in major wireless communication systems, particularly in the context of radar technology [14]. A narrow-band channel is one in which the signal bandwidth does not crucially exceed the coherence bandwidth of the channel. Narrow-band signals for bandpass filters have a small fractional bandwidth, which is given below:

(1) $$\Delta =\frac{f_{H}-f_{L}}{f_{C}}$$

where $f_{H}$ , $f_{L}$ , and $f_{C}$ are the upper cutoff frequency, lower cutoff frequency, and center frequency, respectively.

The low-frequency spectrum of narrow-band signals also assists in minimising the power consumption required to transmit and process the signal.

In radar systems, employing multiple filters for various frequencies incurs space, time, and financial expenses. A preferred solution to address this drawback is the use of a switchable filter bank [15,16,17]. This configuration involves placing two or more filters in close proximity, ensuring high isolation and improved performance. Furthermore, these filters can be tuned to their respective frequencies, offering a more efficient and cost-effective solution [18]. A narrow-band filter was designed at the Ku band with a 0.05% bandwidth and sharp rejection of >48 dB. Technology based on dielectric resonators was utilised in the development of the filter [19]. A highly sensitive and high-rejection narrow bandwidth of 0.5% bandpass filter was designed using stripline technology for nonlinear junction detectors.

In [20], a narrow-band filter using bandpass technology is implemented where negative resistance is used to increase the selectivity and the 3 dB bandwidth of the filter is less than 1%. In [21], the FR4 and Arlon 25N substrates are used in the development of a bandpass filter that makes use of a hairpin resonator structure and operates at a centre frequency of 2.1 GHz. The bandwidth of the filter is 400 MHz, the insertion loss, measured in decibels (IL), is less than 2.5, whereas the return loss, measured in decibels (RL), is greater than 20.

A parallel inductive switch network (PISN) is used to make a re-configurable filter bank with a wide range. Three filters are designed on a single substrate using the lumped element method. Inductive switches that are inductive PIN diodes are used to switch between different frequency filters [22]. Coupled line resonators are used to design a filter bank with three filters from 18 to 40 GHz. MMIC switches are used to switch between the filters. The insertion loss is approximately 12 decibels, whereas the return loss is greater than 60 decibels [23]. A perfect decomposition narrow-band finite impulse response filter bank is designed [24]. The frequency range of this filter bank-based RF transceiver ranges from 470 MHz to 698 MHz. In [25], ceramic resonator technology is used to design four bandpass filters. Dual passband microstrip-based stepped impedance resonators are designed at 2.14 and 4.33 GHz [26]. A tunable filter with 13% relative bandwidth and a frequency range of 18.5 to 21.05 GHz is designed using tapped line feedings. MEMS capacitors are used as capacitive switches [27,28]. The switched filter bank consisting of four filters in the S and C frequency bands is designed using the quarter-wave and stepped impedance technique. MEMS switches are used for switching purposes and switching is controlled using an Atmega-328p controller [29]. An electronically tunable filter is designed at a tunable range of 700 MHz to 1.33 GHz with an insertion loss less than 2 dB. The filter is implemented on a suspended substrate [30,31]. Surface acoustic wave (SAW) bandpass filters are fabricated on a low-temperature co-fired ceramic (LTCC) substrate for ISM band frequency and the insertion loss (IL) is less than 1.5 dB [32]. In [33], two bandpass filters are designed and switched using PIN diodes at the center frequency of 1.955 GHz with 140 MHz bandwidth and at 2.44 GHz with 80 MHz bandwidth. Bandpass microstrip filters are used to reduce harmonics in 5G networks [34,35]. For switching, components like PIN diodes and capacitors are used [36,37,38]. Three pole bandpass filters are designed using microstrip lines on an alumina substrate. An SP3T MEMS switch is employed for switching between filters. The passband range is 14 GHz to 18 GHz, the insertion loss of all filters is less than 2 dB, and return loss is more than 10 dB [39]. Using ohmic and capacitive switches, a unique SP4T RF MEMS device is suggested in [40]. It is made with a silicon substrate with a 1 mm^2^ surface area. This RF switch features an excellent X-band insertion loss of less than 0.7 dB between each port and a strong isolation of 70.64 dB overall. In [41], the notch band is created using resonators coupled to bandpass filters with a stepped impedance resonator. In [42], innovative C-band wide band stop filter topologies with a simplified microstrip implementation are given.

**Figure 1:**
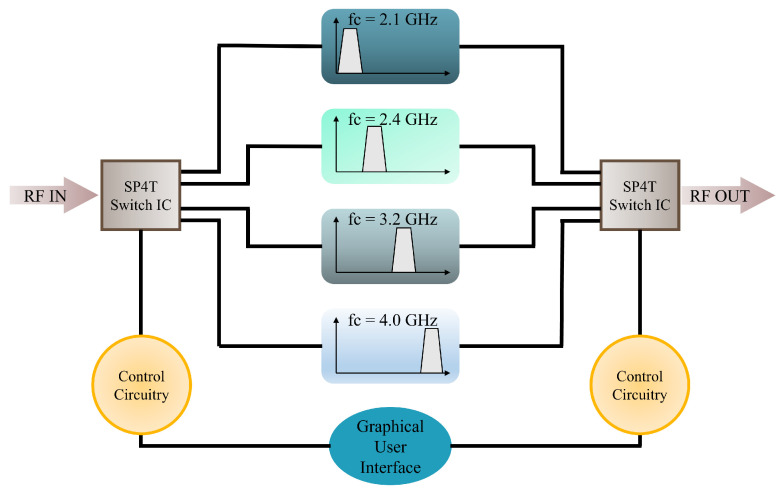
Block diagram of proposed filter bank.

As shown in Figure 1, the switching filter bank is proposed for use with narrow-band filters to increase sensitivity and noise. However, these filter banks require manual operation. If the target’s frequency changes while you are manually monitoring it, the receiver system is unable to keep up, since someone must constantly adjust the system’s frequency to match the target’s new frequency.

**Figure 2:**
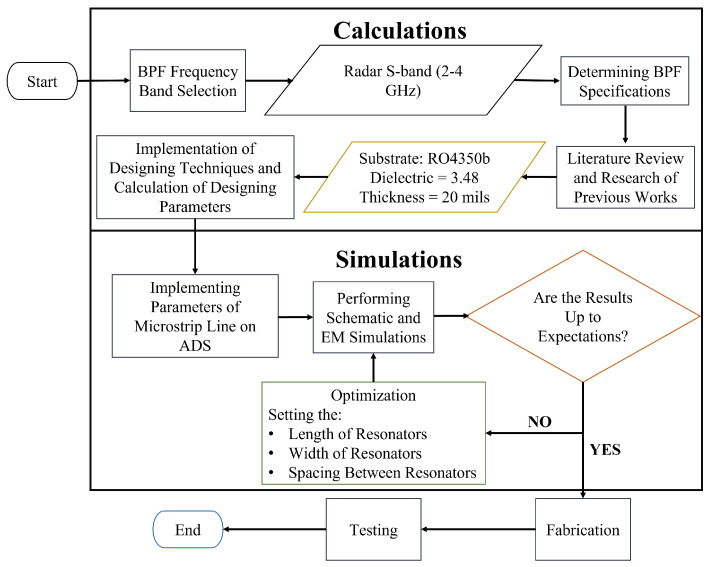
Design methodology for hairpin filter bank.

Hence, to enhance the signal susceptibility, minimise noise, and ensure swift switching without compromising the probability of intercept in the receiving network, we introduce a narrow-band RF switched filter bank using hairpin filters for radar applications. Filter banks are indispensable for maintaining the integrity of backend signal processing. Ineffective noise can undermine the usefulness of the signal, leading to ineffective detection. An optimal filter bank must exhibit zero insertion loss and a return loss exceeding 40 dB for each filter. Rapid switching is imperative to prevent delays, and power consumption should be minimised. The process of designing a hairpin filter bank is depicted in Figure 2.

## 2. Design and Simulation

One method to design a filter bank is utilising lumped element components. However, millimeter waves can be distorted by the lumped components due to parasitic effects. A more favorable solution is to use surface-mounted devices (SMDs), as they lack lead wires and their compact size makes them easier to handle. Another approach involves the use of microstrip-based filters. There are various techniques for designing filters with microstrip technology, such as stepped impedance resonators and coupled resonators, but this specific design employs the hairpin technique. The proposed design includes four bandpass filters integrated with high-switching-speed SP4T MEMS switches. The switches are connected to an external control circuitry that includes Arduino UNO that has microcontroller Atmega 328p installed in it. Low-loss SMA connectors are used to pass signals through the filter bank.

**Figure 3:**
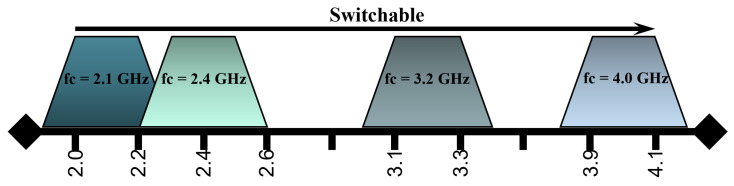
Frequency bands of hairpin-based bandpass filter bank.

The proposed filter bank consists of four filters where the centre frequencies of these bandpass filters are 2.1 GHz, 2.4 GHz, 3.2 GHz, and 4.0 GHz with bandwidths of 9%, 8%, 6%, and 5%, respectively, as illustrated in Figure 3.

These frequency bands include the ISM band and other sub-6 GHz frequencies, which are widely used in both commercial and defence areas.

### Hairpin Filter Design and Fabrication

The design of hairpin filters involves the use of microstrip technology and the unique “hairpin” shape. The basic structure of a hairpin filter consists of a microstrip transmission line that is folded back on itself to form a U-shape. The input port is connected to one end of the transmission line, while the output port is connected to the other end. To tune the filter to the desired frequency response, resonators in the form of stubs are added to the transmission line. The length and impedance of these stubs are calculated precisely to resonate at the target frequency. Multiple stubs can be utilised to achieve a more intricate response if needed. The selectivity and stopband rejection of the filter are determined by its quality factor, which is influenced by the design of the stubs and the microstrip transmission line. Careful attention must be paid to the dimensions and materials used in the design to ensure optimal performance. By combining the unique hairpin shape with microstrip technology and precise resonator design, hairpin filters provide a cost-effective solution for many RF and microwave applications. The size of the microstrip coupled line BPF is large compared to a hairpin filter due to the use of normal $\frac{\lambda }{2}$ resonators. The design principle of the hairpin filter is same as that of half-wave parallel coupled resonators. The only difference is it takes less space, as the resonators are folded in the design. However, this folding leads to shorter coupled line lengths, diminishing the coupling between resonators. The resonator folding introduces coupling capacitance, thereby altering the scattering parameters of a filter and presenting a considerable optimisation challenge. The attenuation rate is contingent on the quantity of hairpin resonators within the filter. The dimensions of transmission lines or microstrip lines in a hairpin filter are dictated by the signal wavelength, and their relative electrical size is determined through a comparison with the wavelength.

For hairpin filters, two types of input structure can be used, namely, coupled line and tapped line. Due to the low spacing at the end of resonators in coupled line input, large coupling appears, which degrades the insertion loss (IL) and the return loss (RL). In contrast, tapped input resonators give flexibility in the tapped input location along with better insertion and return loss. The quality factor and mutual coupling between resonators can be calculated using following equations.

(2) $$Q_{e1}=\frac{g_{0}g_{1}}{FBW}$$

(3) $$Q_{en}=\frac{g_{0}g_{n+1}}{FBW}$$

(4) $$M_{i,i+1}=\frac{FBW}{\sqrt{g_{i}g_{i+1}}}\;i=1,2,\ldots ,n-1$$

where $Q_{e1}$ and $Q_{en}$ represent the external quality factors of resonators at the input and output, respectively, and $M_{\left(i,i+1\right)}$ represents the coupling coefficients between the adjacent resonators.

The standard bandpass filters produce harmonics, which is why the design of microwave bandpass filters starts with designing a low-pass filter, then transforming it into a bandpass filter using numerous calculations. The lumped element bandpass filter is then transformed into half-wave coupled line resonators. Their respective length and widths for inductance and capacitance are calculated using the LineCalc tool in advanced design system (ADS) software. For 0.5 dB equal ripple response, the order of all bandpass filters is the sixth order for significant rejection, i.e., >15 dB. Resonance will occur when both inductive and capacitive reactance become equal.

(5) $$X_{L}=2\pi fL$$

(6) $$X_{C}=\frac{1}{2\pi fC}$$

where *L* and *C* represent inductance and capacitance, respectively; $X_{L}$ represents inductive reactance; $X_{C}$ represents capacitive reactance; and *f* represents frequency.

**Figure 4:**
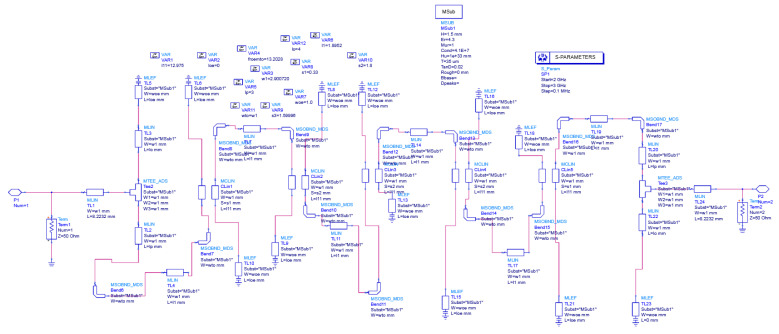
ADS schematic view of hairpin filter.

**Figure 5:**
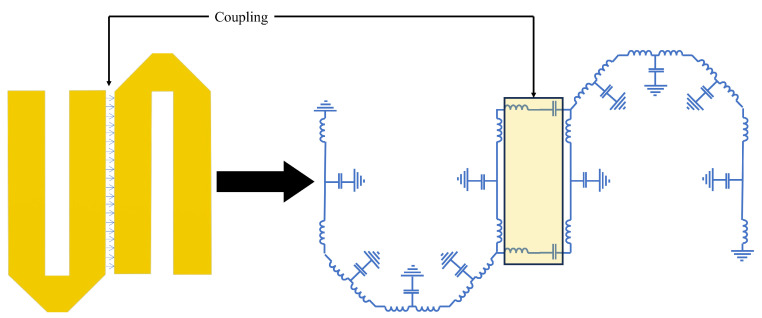
Approximate L-C model of basic hairpin resonator.

**Figure 6:**
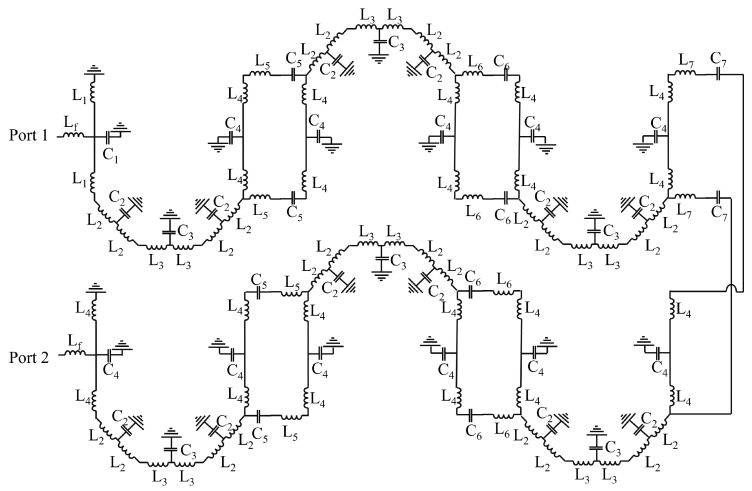
Approximate L-C model of hairpin filter.

The inductor and capacitor values will remain constant, whereas frequency varies the reactance. The schematic of a hairpin filter designed in ADS is shown in Figure 4. Figure 5 illustrates the approximate equivalent L-C model for the fundamental hairpin resonator, while Figure 6 displays the approximate equivalent circuit for the entire hairpin filter [43,44]. It is important to emphasise that this LC model is an approximation serving solely to describe the microstrip resonator’s behavior and is not capable of capturing all the intricacies of its response. The interaction between two resonators is represented by a series capacitor and inductor. Additionally, a T-shaped model is employed to characterise each side of the hairpin resonator, incorporating two inductors $\left(L_{R}\right)$ and one grounded capacitor $\left(C_{R}\right)$ . Table 1 provides the values of inductors and capacitors corresponding to each hairpin filter.

**Figure 7:**
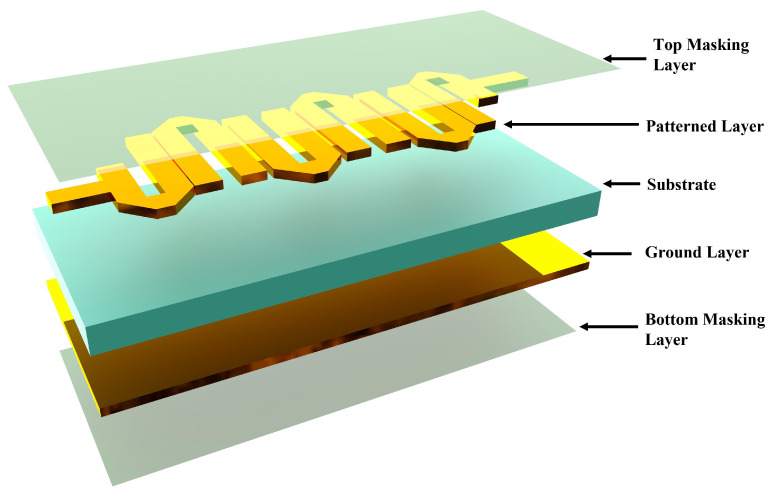
Three-dimensional configuration of proposed hairpin filter.

The design presented in Figure 7 features a compact filter utilising a three-dimensional stacking arrangement. It consists of five layers, ranging from a top masking layer to a bottom masking layer, with two metal layers in between. The top metal layer features a patterned hairpin design, while the bottom layer serves as a ground plane. Upon completion of the PCB manufacturing process, the copper traces on the board are prone to oxidation and corrosion due to exposure to the environment. To prevent these issues and prolong the life of the PCB, a protective covering known as a solder mask layer is applied. This not only prevents oxidation but also shields the PCB from environmental factors like dirt and pollutants that could cause shorts over time.

To build the switchable filter, four hairpin BPFs are placed on a single substrate, along with SP4T switches used throughout the switching process. The substrate used for the hairpin filter design is Rogers’ RO4350b, which has negligible dielectric losses at gigahertz frequencies, and its specifications are listed in Table 2. Solid-state switches are employed for switching purposes. An SP4T switch from mini circuits with model number HSWA4-63DR+ is integrated. It is a low-loss, high-speed, and high-isolation switch.

**Figure 8:**
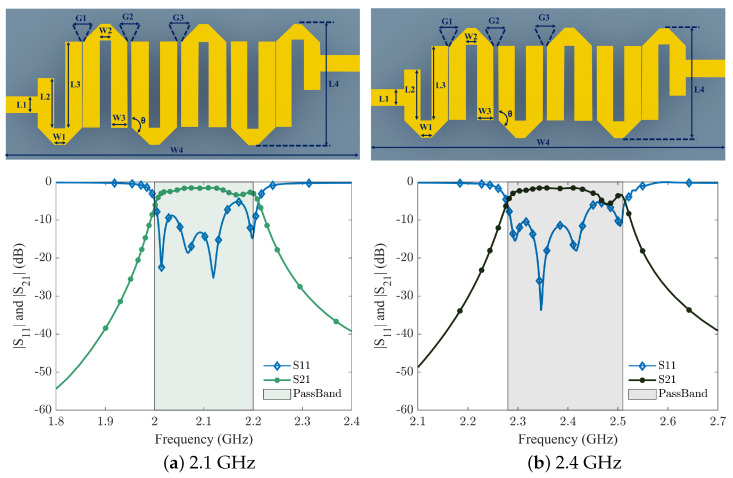
Design layout of 2.1 GHz and 2.4 GHz hairpin filter and its simulated results.

**Figure 9:**
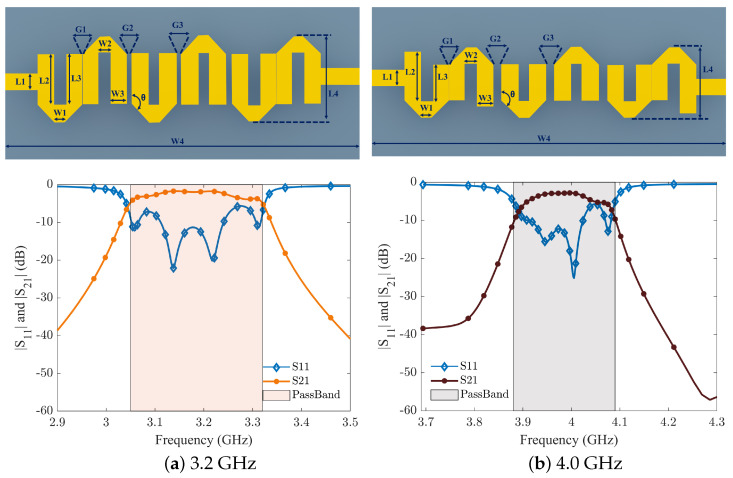
Design layout of 3.2 GHz and 4.0 GHz hairpin filter and its simulated results.

Figure 8 and Figure 9 show the layouts and S-parameter responses of hairpin bandpass filters, which are simulated in an advanced design system (ADS). The layout dimensions for all four proposed filters are mentioned in Table 3. In Figure 8a, the simulation results of BPF1 (2.0–2.2 GHz) along with its layout are shown. BPF1 features a peak insertion loss (IL) of approximately 0.9 dB, with the minimum return loss (RL) below 25 dB in the band. Similarly, Figure 8b illustrates that BPF2 (2.3–2.5 GHz) has a maximum IL of 0.85 dB, accompanied by an acceptable RL.

The insertion loss (IL) and return loss (RL) characteristics of the third hairpin-based bandpass filter, denoted as BPF3 (3.05–3.3 GHz), are depicted in Figure 9a. It shares the same maximum IL as BPF2, with the minimum RL falling below 20 dB within the designated frequency band. Moving on to the s-parameter outcomes for BPF4, illustrated in Figure 9b, the maximum IL is recorded at 1.6 dB, while the minimum RL remains below 20 dB. Since all of the filter frequencies are apart, there is already a significant level of isolation between the four filters. To be on the safe side, the attenuation is set to be greater than 40 dB after determining the relevant fractional bandwidth of each filter. Each filter’s insertion loss is kept in simulation close to zero so that, after integrating switches, their loss can be taken into account to prevent the signal’s power loss.

Additionally, each filter’s dimensions are displayed alongside the results. Every filter has an inverse relationship between frequency and filter size. Each hairpin is assumed to bend at a 135° angle. The majority of the filter bank’s dimensions remain constant; however, changing certain of them has a significant impact on the frequency and IL of the corresponding filter. After calculations, not all dimensions are accurate. The calculated parameters were entered into ADS and subsequently tuned for the optimum outcomes based on the results. Therefore, optimisation was crucially important in obtaining the results that are displayed.

For radar applications, the frequencies for this filter bank are assumed to be in the S-band. This demonstrates that high power is required on the transmitting side of radar for signal transmission, whereas on the receiving side, the radar receives the high-power signal and passes it through the LNA, which lowers the signal’s power before it passes through the filter bank, making it the ideal device for use at the receiving side. On the other hand, high-power filters are needed at the transmitting backend, where waveguides can be employed as high-power filters. The strength of the signal is the first factor to be taken into account while discussing radar. When the IL is 3 dB or higher, the signal will continue to be at half strength or less. This means that in order to comprehend the signal, the IL must be lower than 3 dB. The power of the signal remains over 50% while being filtered out, as shown in the simulated results of the proposed filters.

**Figure 10:**
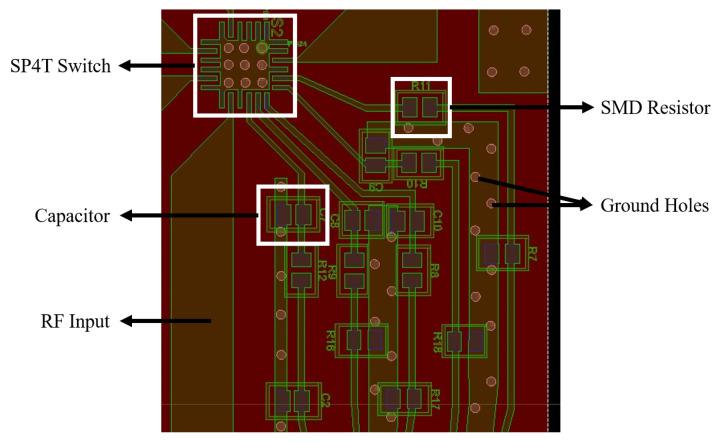
Layout of SP4T switch and SMD components.

After simulating all four filters according to the requirements, the next step was to design the layout of SP4T switches, and the specification of the switch is already mentioned in Table 2. The layout of the SP4T switch can be seen in Figure 10. There are twenty-four total pins on it, five of which are used for input and output, three for control, one for operating voltage, and one for ground. The other pins are all grounded as well.

**Figure 11:**
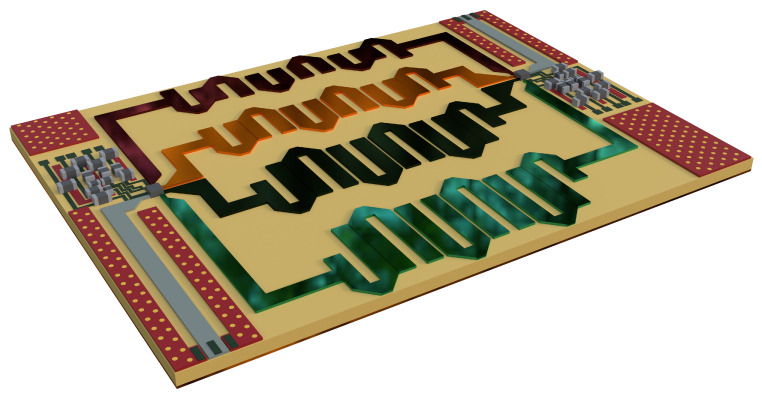
Three-dimensional layout of designed RF switched filter bank.

There are a total of four states that the system can provide: channel 1 enabled only (00), channel 2 enabled only (01), channel 3 enabled only (10), and channel 4 enabled only (11). Some capacitors and resistors are used at the control and voltage pins according to the characterisation circuit of the switch. The list of SMD components integrated with it is shown in Table 4. The layouts of all four bandpass filters were then integrated together on a single substrate along with two SP4T switches, shown in Figure 11. The total size of the filter bank is 129.579 mm × 99.916 mm.

## 3. Implementation and Results

**Figure 12:**
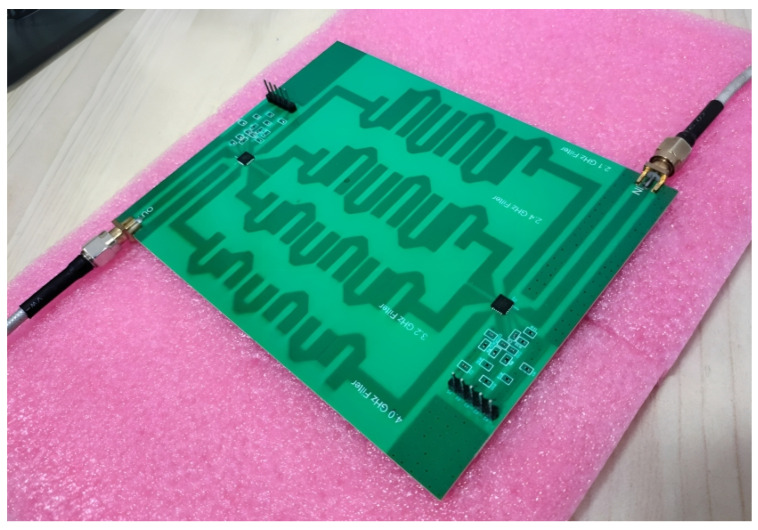
Fabricated RF switched filter bank.

The filter bank was fabricated on Rogers’ RO4350b substrate and all SMD components, which includes resistors and capacitors, were soldered as shown in Figure 12 and tested using a vector network analyzer (VNA). The SMA connectors that are used have an operating frequency of greater than 1 GHz. The testing setup consists of an Arduino UNO, VNA, and coaxial cables.

**Figure 13:**
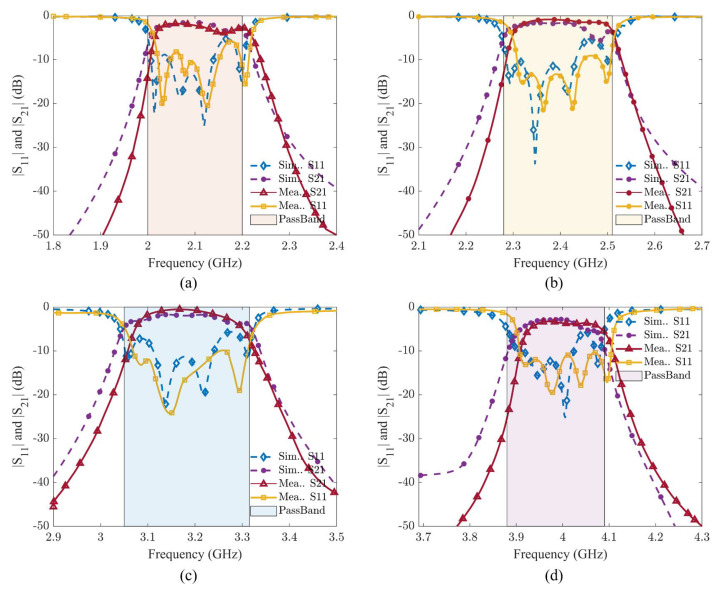
Simulated and measured S11 and S21 of filter bank. (**a**) 2.0–2.2 GHz. (**b**) 2.3–2.5 GHz. (**c**) 3.1–3.3 GHz. (**d**) 3.9–4.1 GHz.

Figure 13 shows the comparison of simulated and measured results for all four filters, which shows that these measured results are in good agreement with the simulated one. In Figure 13a, the outcomes for BPF1 are presented. For BPF1, the maximum measured insertion loss (IL) remains below 1.8 dB, while the minimum return loss (RL) exceeds 20 dB, and the 3 dB bandwidth spans 210 MHz. Moving to Figure 13b, the results for BPF2 indicate a maximum measured IL of 1.2 dB, with a minimum RL exceeding 15 dB. The 3 dB bandwidth for BPF2 is approximately 215 MHz. In Figure 13c, the maximum IL and minimum RL for BPF3 are less than 1.4 dB and greater than 15 dB, respectively. BPF3’s 3 dB bandwidth is measured at 240 MHz. Finally, Figure 13d displays the maximum insertion loss and minimum return loss for BPF4, recorded at 2.8 dB and 21 dB, respectively, with a 3 dB bandwidth of 190 MHz. The filter bank is working perfectly and showing good isolation between filters on switching the channels through four different combinations applied using the Arduino controller. We have tested the functionality of the filter bank by sending audio signals at centre frequencies using two USRPs and antennas, one at the input side as a transmitter and other at the output side of the filter bank as a receiver.

In the end, we have summarised in Table 5 some recent studies that reported on filter banks and compared them with our own work.

## 4. Conclusions

A high-performance, low-cost, and fully integrated bandpass filter bank based on hairpin topology is presented in this paper. SP4T switches for switching between different frequency bands are used, which provide high isolation and low loss. The overall size-reduction objective along with low insertion loss is achieved successfully. The entire design analysis of the filter bank is performed in ADS software. All four filters have a low insertion loss and good stopband performance. The measured insertion loss (IL) and return loss (RL) for all four filters are in good agreement with the simulated one. The overall size of the filter bank is 129.5 mm by 99.9 mm.

## Figures and Tables

**Table 1:** LC values of given equivalent circuit.

	BPF 1	BPF 2	BPF 3	BPF 4
Centre Frequency	2.1 GHz	2.4 GHz	3.2 GHz	4.0 GHz
$L_{f}$	1.33 nH	1.33 nH	1.33 nH	1.33 nH
$L_{1}$	0.70 nH	0.70 nH	0.70 nH	0.70 nH
$L_{2}$	0.48 nH	0.48 nH	0.48 nH	0.48 nH
$L_{3}$	0.12 nH	0.12 nH	0.12 nH	0.12 nH
$L_{4}$	1.20 nH	1.02 nH	0.69 nH	0.50 nH
$C_{1}$	2.72 pF	2.72 pF	2.72 pF	2.72 pF
$C_{2}$	0.94 pF	0.94 pF	0.94 pF	0.94 pF
$C_{3}$	0.47 pF	0.47 pF	0.47 pF	0.47 pF
$C_{4}$	4.61 pF	3.93 pF	2.68 pF	1.94 pF

**Table 2:** SP4T switch and substrate specifications.

SP4T Switch Specifications		Substrate Specifications	
Switch Model	HSWA4-63DR+	Substrate	Rogers’ RO4350b
Isolation (0.9 GHz)	61 dB	Dielectric Constant	3.48
Insertion Loss (0.9 GHz)	0.9 dB	Substrate Thickness	0.508 mm (20 mils)
Switching Speed	255 ns	Loss Tangent	0.0037
Rise Time	100 ns	Metal Thickness	0.035 mm
Fall Time	100 ns	-	-
Operating Temperature	−40 °C to 105 °C	-	-
RF Power Input	34 dBm	-	-

**Table 3:** Physical dimensions (in mm) of all four filters.

	BPF 1	BPF 2	BPF 3	BPF 4
Centre Frequency (GHz)	2.1	2.4	3.2	4.0
*L*_1_	3.41	3.41	3.41	3.41
*L*_2_	10.77	10.76	10.77	10.77
*L*_3_	18.23	15.88	10.96	7.67
*L*_4_	25.77	23.07	18.16	15.23
*W*_1_	1.89	1.89	1.89	1.89
*W*_2_	2.59	2.59	2.59	2.59
*W*_3_	3.41	3.41	3.42	3.42
*W*_4_	60.31	60.39	60.56	62.66
*G*_1_	0.14	0.15	0.14	0.10
*G*_2_	1.15	1.17	1.20	1.85
*G*_3_	1.15	1.16	1.19	1.85
θ	135°	135°	135°	135°

**Table 4:** SMD components and their values.

Components	Value
C2	1 uF
C7, C8, C9, C10	100 pF
R7, R8, R9, R10, R11, R12	0 M Ω
R16, R17, R18	1 M Ω
S2	HSWA4-63DR+

**Table 5:** Literature survey of previous studies.

References	[45]	[46]	[47]	[48]	[22]	[49]	[50]	Current Work
Centre Frequencies (GHz)	0.4–3.0	UWB 2.4	4.5	14.25 16.25	0.040, 0.0675, 0.1135, 0.189, 0.315, 0.453	2.335, 2.425, 2.550	0.600, 0.685, 0.770, 0.850	2.1, 2.4, 3.2, 4.0
Insertion Loss	3.2–6.8 dB	1.9–2.1 dB	0.8 dB (simulated)	2 dB	0.1 dB (simulated)	4–6 dB	3.5–4 dB	1.2–3.0 dB
Return Loss	12–38 dB	18 dB	20 dB	15 dB	16 dB	11–16 dB	25 dB	15–25 dB
Relative Bandwidth	450	400	5.2%	500 MHz	20–158 MHz	6–8.5%	11–16%	200 MHz
Size ( $\lambda_{g}$ × $\lambda_{g}$ )	0.034 × 0.048	0.049 × 0.049	NA	NA	NA	0.763 × 0.389	0.500 × 0.290	0.903 × 0.693
Topology	Nested Filters	Half-wave and quarter-wave resonators	Hairpin	Waveguide	Lumped Element	Hairpin Comb	Hairpin	Hairpin
Switching Technique	MEMS Switches	PIN diode	MEMS Switches	MEMS Switches	Not selected (no hardware)	Serial Switched	MMIC	Solid-State Switches
Channels	2	2	2	2	6	3	4	4

## Data Availability

Data is contained within the article.
